# Thermal Decomposition of Nanostructured Bismuth Subcarbonate

**DOI:** 10.3390/ma13194287

**Published:** 2020-09-25

**Authors:** Su Sheng, Shengming Jin, Kuixin Cui

**Affiliations:** 1 School of Minerals Processing and Bioengineering, Central South University, Changsha 410083, China; shengsu0101@163.com (S.S.); shmjin@csu.edu.cn (S.J.); 2 Key Laboratory for Mineral Materials and Application of Hunan Province, Central South University, Changsha 410083, China

**Keywords:** bismuth subcarbonate, thermogravimetric analysis, thermal decomposition kinetics

## Abstract

Nanostructured (BiO)_2_CO_3_ samples were prepared, and their thermal decomposition behaviors were investigated by thermogravimetric analysis under atmospheric conditions. The method of preparation and Ca^2+^ doping could affect the morphologies of products and quantity of defects, resulting in different thermal decomposition mechanisms. The (BiO)_2_CO_3_ nanoplates decomposed at 300–500 °C with an activation energy of 160–170 kJ/mol. Two temperature zones existed in the thermal decomposition of (BiO)_2_CO_3_ and Ca-(BiO)_2_CO_3_ nanowires. The first one was caused by the decomposition of (BiO)_4_(OH)_2_CO_3_ impurities and (BiO)_2_CO_3_ with surface defects, with an activation energy of 118–223 kJ/mol, whereas the second one was attributed to the decomposition of (BiO)_2_CO_3_ in the core of nanowires, with an activation energy of 230–270 kJ/mol for the core of (BiO)_2_CO_3_ nanowires and 210–223 kJ/mol for the core of Ca-(BiO)_2_CO_3_ nanowires. Introducing Ca^2+^ ions into (BiO)_2_CO_3_ nanowires improved their thermal stability and accelerated the decomposition of (BiO)_2_CO_3_ in the decomposition zone.

## 1. Introduction

Ternary bismuth-containing compounds have attracted remarkable attention owing to their desirable properties [1,2,3,4,5,6,7]. In particular, (BiO)_2_CO_3_ has a typical Sillén structure and very high stability at oxidizing environments, wherein the Bi–O layers and CO_3_^2−^ layers are intergrown with a plane of the CO_3_^2−^ group orthogonal to the Bi–O layer [8]. Given its unique layered structure, suitable band gap, and high stability, (BiO)_2_CO_3_ is a promising candidate for photocatalysts [9,10,11], antimicrobial agents [12], cholesterol biosensors [13], and humidity sensors [14]. However, (BiO)_2_CO_3_ has a considerably high density, resulting in a relatively lower specific surface area than other photocatalysts or antimicrobial agents. Thus, uniformly dispersing (BiO)_2_CO_3_ during its practical application and ensuring sufficient contact with targets are difficult. Moreover, the band gap of (BiO)_2_CO_3_, which depends on its morphology and size distribution, is the most important parameter in the photocatalytic field [15]. The smaller the size, the narrower the band gap. For instance, (BiO)_2_CO_3_ nanotubes with an average diameter of 7 nm possess a band gap of 3.00 eV, whereas (BiO)_2_CO_3_ nanoplates with a thickness of 70–80 nm have a band gap of 3.39 eV [16]. The most common method of increasing specific surface area and decreasing band gap is to prepare nanoscale (BiO)_2_CO_3_ materials. Zhao et al. reported that the BET specific surface area and band gap of (BiO)_2_CO_3_ powders vary along the (BiO)_2_CO_3_ morphologies [17]. For example, the BET specific surface area and band gap of sponge-like microspheres are 43.99 m^2^/g and 2.87 eV, respectively; however, the corresponding values for plate-like microspheres are 38.86 m^2^/g and 3.34 eV.

Nanomaterials generally possess poor thermal stability compared to their bulk counterparts because of their high specific surface area, quantities of active sites, and large velocity of mass and heat transfer presented at the reaction interface. Thermal performance is an important property for practical storage and application of nanomaterials [18]. Investigating the thermal stability and thermal decomposition kinetics is important for the deeper understanding of the Bi_2_O_2_CO_3_ structure and its practical application. Recently, studies about the thermal treatment of Bi_2_O_2_CO_3_ have been reported. For instance, Pan’s work found that Bi_2_O_2_CO_3_ nanoflowers decomposed to form Bi_2_O_3_/Bi_2_O_2.33_@Bi_2_O_2_CO_3_ composites slowly in air [19]. Another work showed that the α-Bi_2_O_3_/(BiO)_2_CO_3_ heterojunction nanoplate could be obtained by in situ thermal treatment of (BiO)_2_CO_3_ nanoplates [20]. Moreover, β- and α-phase porous Bi_2_O_3_ microspheres have been synthesized by thermal treatment of Bi_2_O_2_CO_3_ microspheres in an air atmosphere [21]. However, the thermal decomposition kinetics of nanostructured (BiO)_2_CO_3_ is rarely reported.

The present work aimed to investigate the effect of structure, preparation method, and doping on the thermal performance and decomposition kinetics of nanostructured (BiO)_2_CO_3_. Thus, (BiO)_2_CO_3_ samples were prepared, and their thermal decomposition behaviors were investigated in detail by thermogravimetric analysis under atmospheric conditions. 

## 2. Materials and Methods

### 2.1. Materials

Na_2_CO_3_, NaCl, and CaCl_2_ (Analytical grade, Xilong Chemical Co., Ltd., Guangdong, China), were used without further purification. β-Bi_2_O_3_ powders were obtained from Changde Fine Chemical Co. Ltd. (Hunan, China).

### 2.2. Preparation and Characterization of Bismuth Carbonate

In a typical synthesis procedure of (BiO)_2_CO_3_ nanowires (marked as BCO), NaCl (2.338 g) and Na_2_CO_3_ (0.212 g) were dissolved in 70 mL of deionized water. The pH of the solution was adjusted to 3.0 using 1 M HCl solution, and then β-Bi_2_O_3_ powders (0.932 g) were added into the solution. The mixture was then transferred into a 100 mL Teflon-lined stainless-steel autoclave, magnetically stirred at 160 °C for 6 h, and subsequently cooled to room temperature. Products were collected by filtration, washed with deionized water and ethanol several times, and dried overnight at 60 °C. Ca–(BiO)_2_CO_3_ nanowires (marked as Ca–BCO) were prepared through adding 0.022 g of CaCl_2_ during the synthesis of (BiO)_2_CO_3_ nanowires. For comparison, (BiO)_2_CO_3_ nanoplates (marked as C–BCO) were prepared using Bi(NO_3_)_3_∙5H_2_O and Na_2_CO_3_ in the following procedure: Bi(NO_3_)_3_∙5H_2_O (0.96 g) was first dissolved in dilute HNO_3_ (1 M, 5 mL) under continuous stirring. Once the above solution became clear, it was added dropwise to an aqueous solution of Na_2_CO_3_ (0.2 M, 50 mL), and plenty of white precipitates formed. The suspension was further stirred for 30 min at 55 °C. The products were collected, washed with deionized water, and dried overnight at 60 °C.

The crystal phase and composition of as-prepared products were analyzed using an X-ray powder diffractometer (XRD: D/max 2550, Rigaku, Tokyo, Japan) with Cu-Kα irradiation (λ = 0.1548 nm) at a scanning step of 10°/min at 10–70° (2θ). Field emission scanning electron microscopy (FE-SEM: FEI Nova NanoSEM 230, with an accelerating voltage of 10 kV), transmission electron microscopy (TEM), and high-resolution TEM (HRTEM) were used to characterize the morphology, structure, and grain size of the obtained products. The thermal stability was examined through thermogravimetric (TG) analysis using a Netzsch STA 449C thermo-analyzer (Netzsch, Selb, Germany) with heating rates of 5, 10, 15, and 20 °C/min from 40 to 700 °C under atmospheric conditions.

### 2.3. Thermal Decomposition Kinetics Model

Thermal decomposition kinetics of all samples was studied based on the TG data. Equation (1) is the basic kinetics equation [22].

(1) $$\frac{d\alpha }{{\left(1-\alpha \right)}^{n}}=\frac{A}{\beta }\exp(-\frac{E_{a}}{RT})dT$$

where *A* and *E_a_* are the pre-exponential factor and the apparent activation energy, respectively, T is the temperature, α is the extent of conversion, *n* is the reaction order, *β* is the heating rate, and R is the gas constant.

Obviously, Equation (1) does not have an analytical solution independently. Many works have been done to obtain reasonable kinetic parameters, including differential methods and integral methods. Among a number of differential methods, the widest used one is the Kissinger equation [23,24,25]. In this case, the activation energy is calculated from the *T_max_* where the maximum decomposition rate occurs at different heating rates. The maximum decomposition rate occurs when *dα/dt* = 0. Thus, differentiating Equation (1) with respect to time and equating the resulting expression to zero lead to the following equation:

(2) $${\left(\frac{d^{2}\alpha }{dT^{2}}\right)}_{max}=\left(\frac{d\alpha }{dT}\right){\left[\frac{E_{a}}{RT_{max}^{2}}-\frac{nA}{\beta }exp\left(-\frac{E_{a}}{RT_{max}}\right){\left(1-\alpha \right)}_{max}^{n-1}\right]}_{max}$$

Kissinger assumed the product of ${\left(1-\alpha \right)}_{max}^{n-1}$ = 1 and that it is independent of the heating rate. In such a case, the logarithmic expression of Equation (2) can be written:

(3) $$ln\frac{\beta }{T_{max}^{2}}=-\frac{E_{a}}{R}\left(\frac{1}{T_{max}}\right)+ln\frac{AR}{E}$$

Thus, the activation energy can be computed from the linear dependence of ln(*β*/*T_max_*^2^) on 1/*T_max_* at various heating rates.

Among all the integral methods, the relative accurate approximation by Murray and White yields the Kissinger–Akahira–Sunose equation [22,26]:

(4) $$ln(\frac{\beta }{T_{\alpha }^{2}})=C-\frac{E_{a}}{RT_{\alpha }}$$

where *C* is a constant at a given conversion, *α*. Thus, at a given heating rate *β*, one can find a particular *α* and a corresponding temperature *T*. At a given *α*, by varying *β*, one can find the corresponding *T* that is a function of *β*. Hence, if a plot of ln(*β/T*^2^) versus 1/*T_α_* is linear, the activation energy *E_a_* can be calculated from the slope of *E_a_*/R.

## 3. Results

### 3.1. Characterization of (BiO)_2_CO_3_ Samples

**Figure 1:**
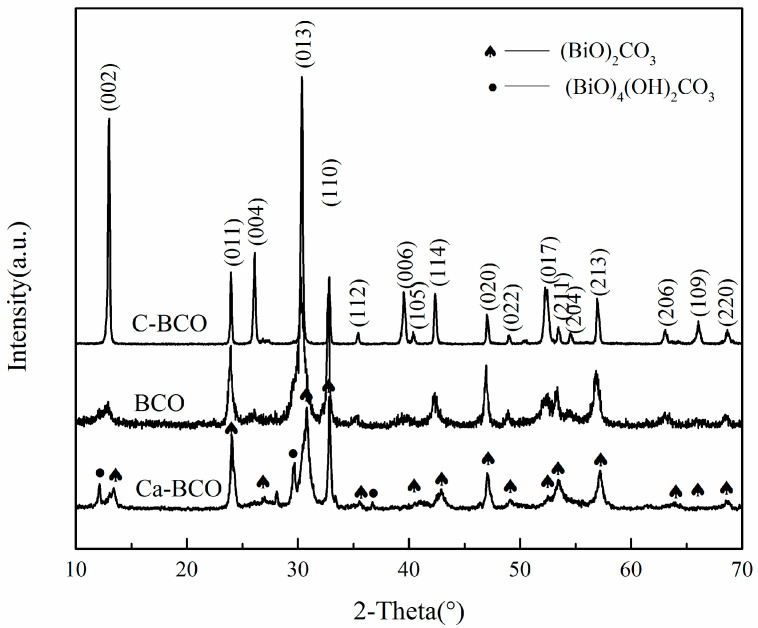
XRD patterns of as-synthesized (BiO)_2_CO_3_ nanoplates (C-BCO), as-synthesized (BiO)_2_CO_3_ nanowires (BCO), and as-synthesized Ca-(BiO)_2_CO_3_ nanowires (Ca-BCO).

Figure 1 shows the XRD patterns of the as-prepared (BiO)_2_CO_3_ nanoplates, nanowires, and Ca–(BiO)_2_CO_3_ nanowires. All diffraction peaks of the (BiO)_2_CO_3_ sample obtained from Bi(NO_3_)_3_∙5H_2_O (marked C–BCO) could be readily indexed to an orthorhombic (BiO)_2_CO_3_ with cell parameters a = 3.865 Å, b = 3.862 Å, and c = 13.675 Å (ICDD Card No. 97–009–4740). No peaks of impurities were observed, indicating the high phase purity of products. As for (BiO)_2_CO_3_ nanowires without the addition of CaCl_2_, all the main diffraction peaks could also be indexed to (BiO)_2_CO_3_ (marked as BCO). However, the diffraction intensity was much weaker than that of C–BCO, suggesting that the as-prepared nanowires had a poor crystallinity. Two additional peaks were ascribed to the (BiO)_4_(OH)_2_CO_3_ phase emerged at 2θ ≈ 12.2 and 29.7 (ICDD Card No. 00–038–0579) [27]. Moreover, the (002), (004), and (006) crystallographic planes of the as-prepared product presented broader diffraction peaks compared to the counterparts of C–BCO, which could result from a smaller size along the c-axis. By contrast, when CaCl_2_ was added in the reaction solution, additional diffraction peaks belonging to (BiO)_4_(OH)_2_CO_3_ at 2θ = 12.208, 29.718, and 36.788 (marked as solid circles) were clearly observed in addition to characteristic peaks of the pure (BiO)_2_CO_3_ phase. In addition, (002), (011), and (013) crystallographic planes ascribed to orthorhombic (BiO)_2_CO_3_ shifted to a high angle, suggesting that their corresponding d-value decreased and lattice distortion occurred because Ca^2+^ ions were introduced into the (BiO)_2_CO_3_ crystal.

**Figure 2:**
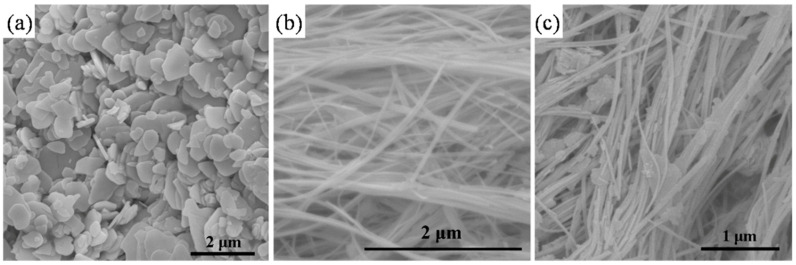
SEM images of C-BCO (**a**), BCO (**b**), and Ca-BCO nanowires (**c**).

The morphologies of the obtained samples were characterized by SEM (shown in Figure 2). Figure 2a shows that nanoplates obtained from Bi(NO_3_)_3_∙5H_2_O were of different size ranges, 0.5–1.5 μm in width and approximately 100 nm in thickness. Figure 2b, on the other hand, shows that the pure (BiO)_2_CO_3_ samples from the hydrothermal method were wire-like nanostructures with a length of tens of micrometers. When Ca^2+^ ions were added into the synthesis system, the main morphologies of Ca–(BiO)_2_CO_3_ remained as a wire-like shape, along with a few nanoplates (Figure 2c).

### 3.2. Thermal Decomposition Characteristics of Nanostructured (BiO)_2_CO_3_

Characteristic temperatures of all samples at every heating rate were determined from the TG and DTG (derivative thermogravimetric analysis) curves (TG–DTG curves are shown in Figures S1–S3). The extrapolated onset temperature of decomposition was obtained by extrapolating the slope of the DTG curve down to the zero level of the DTG axis. The peak temperature was determined using the DTG peak where the maximum decomposition rate was obtained. Obvious differences were presented among the TG-DTG curves of C-BCO, BCO, and Ca-BCO. Two mass loss zones appeared in TG curves of BCO and Ca-BCO while one mass loss zone existed on that of C-BCO. Table 1 lists TG results of three samples.

Results showed that the peak temperature increased with increasing heating rate (Table 1). Only one mass loss range between approximately 320 and 520 °C existed on each TG–DTG curve of C–BCO, indicating that the phase transformation from (BiO)_2_CO_3_ to Bi_2_O_3_ occurred according to the mass loss of 8.33%, which was 8.62% theoretically. The decomposition equation is as follows:

(5) $${\left(\mathrm{BiO}\right)}_{2}{\mathrm{CO}}_{3}\to {\mathrm{Bi}}_{2}O_{3}+{\mathrm{CO}}_{2}$$

For the as-prepared BCO and Ca–BCO, each one has two similar and independent mass loss zones. The first zone between approximately 200 and 380 °C was caused by the decomposition of (BiO)_4_(OH)_2_CO_3_ impurities and defects on the surface of nanowires. The second zone occurred between approximately 380 and 600 °C because of the decomposition reaction of (BiO)_2_CO_3_ in the core of nanowires. When the temperature exceeded 600 °C, the residual weight changed slightly. The thermal decomposition route was summarized in the following reaction sequence [28,29]:

(6) $${\left(\mathrm{BiO}\right)}_{4}{\left(\mathrm{OH}\right)}_{2}{\mathrm{CO}}_{3}\overset{250-380\;{}^{\circ}C}{\to }{2\mathrm{Bi}}_{2}O_{3}+{\mathrm{CO}}_{2}+H_{2}O$$

(7) $${\left(\mathrm{BiO}\right)}_{2}{\mathrm{CO}}_{3}\to {\mathrm{Bi}}_{2}O_{3}+{\mathrm{CO}}_{2}$$

Comparing the peak temperature of C–BCO with those of BCO and Ca–BCO in the second mass loss zone at the same heating rate, the peak temperature was found to increase in the order of C–BCO < BCO < Ca–BCO, indicating an improvement in the thermal stability of nanostructured (BiO)_2_CO_3_. Given that C-BCO was prepared by a co-precipitation method under 55 °C, while BCO and Ca-BCO were prepared by a hydrothermal method under 160 °C, it is believed that the high temperature and pressure are beneficial to the formation of stable (BiO)_2_CO_3_. Correspondingly, the peak temperature of BCO is lower than that of Ca–BCO in the first mass loss zone, indicating that introducing Ca^2+^ ions improved the stability of surface (BiO)_2_CO_3_ nanowires.

### 3.3. Thermal Decomposition Kinetics

**Figure 3:**
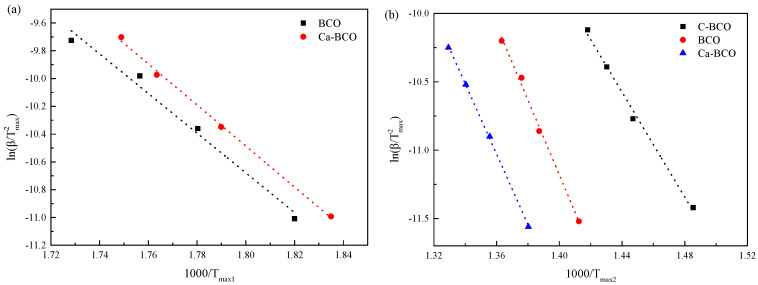
Plots of ln(β/*T_max_*^2^) versus 1000/*T_max_* of (BiO)_2_CO_3_ samples for the first mass loss zone (**a**) and the main mass loss zone (**b**) based on Kissinger’s method.

Figure 3 shows the plots based on Kissinger’s method for the first mass loss zones of as-prepared nanowires (Figure 3a), and the main mass loss zones of three nanostructured (BiO)_2_CO_3_ samples (Figure 3b). The slopes of dotted lines drawn through these plots equal *E_a_*/R such that activation energies *E_a_* were determined. The calculated apparent energies are listed in Table 2. 

**Figure 4:**
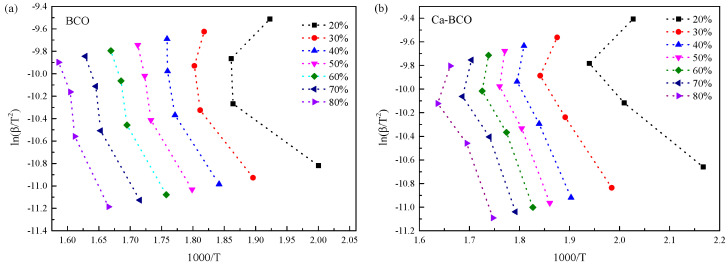
Plots of ln(*β*/*T*^2^) versus 1000/*T* at various mass losses of the first mass loss zone for as-prepared (BiO)_2_CO_3_ nanowires (**a**) and Ca-(BiO)_2_CO_3_ nanowires (**b**) based on the Kissinger–Akahira–Sunose method.

**Figure 5:**
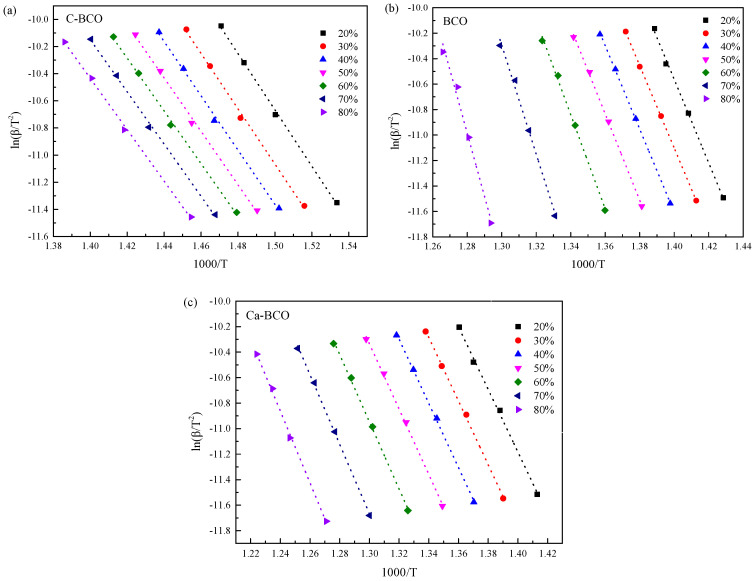
Plots of ln(β/T^2^) versus 1000/T at various conversions of the main mass loss zone for C-BCO (**a**), as-prepared BCO nanowires (**b**), and Ca-BCO nanowires (**c**) based on the Kissinger–Akahira–Sunose method.

Considering that Kissinger’s method is a special case in determining *E_a_,* it may not display the overall trend of *E_a_*. The activation energies of thermal decomposition for nanostructured (BiO)_2_CO_3_ samples were also studied using the Kissinger–Akahira–Sunose method. ln(*β*/*T*^2^) was plotted against 1000/*T* for the first mass loss zone and the main mass loss zone according to Equation (4) in Figure 4 and Figure 5, respectively, to obtain *E_a_*. Each fitted line in Figure 4 and Figure 5 should be straight and parallel to each other in order to give a constant activation energy *E_a_*. However, all curves in Figure 4 are approximately parallel with each other, but are not straight lines, especially for the lower conversions. This behavior indicates that the as-prepared samples underwent a complicated thermal decomposition process. On the one hand, the aforementioned XRD results demonstrated that (BiO)_4_(OH)_2_CO_3_ impurities emerged in the as-prepared BCO and Ca-BCO samples, which decomposed into Bi_4_O_5_CO_3_ and H_2_O between 230 and 325 °C, and then Bi_4_O_5_CO_3_ decomposed into Bi_2_O_3_ and CO_2_ [26]. On the other hand, the surface defects of (BiO)_2_CO_3_ nanowires (shown on the HRTEM image in Figure S4, Supporting Information) make them more active, resulting in a lower temperature limit for decomposition reaction. By contrast, Figure 5 showed that the data points in the second mass loss zone can be approximately fitted to straight lines with negative slopes, and are nearly parallel to each other under different conversion rates. This finding demonstrated that the main mass loss of BCO and Ca-BCO was caused by the decomposition of (BiO)_2_CO_3_ in the cores of nanowires with a single decomposition reaction mechanism. *E_a_* could be calculated and averaged from the slopes. Results for *E_a_* are shown in Table 3.

Table 3 shows that the calculated apparent activation energy of C–BCO decreased from 178.49 to 157.42 kJ/mol when the conversion rate increased from 20 to 80%. The apparent activation energies of BCO and Ca–BCO increased from 246.71 to 334.49 kJ/mol and 204.02 to 234.47 kJ/mol, respectively. The values of C–BCO and Ca–BCO obtained using the Kissinger–Akahira–Sunose methods are comparable to those calculated by Kissinger’s methods, but the former is quite higher than the latter, especially at a high conversion rate. Different kinetic analysis methods are complimentary, as suggested by the ICTAC Kinetics Project [22]. Therefore, an appropriate apparent activation energy range should be obtained by combining all observations in Table 2 and Table 3, as well as Figure 3 and Figure 5. Consequently, a general activation energy range of 160–170 kJ/mol was suggested for C–BCO, 230–270 kJ/mol for BCO, and 210–223 kJ/mol for Ca–BCO. The calculated apparent activity energies of as-prepared nanowires were interestingly higher than those of as-prepared nanoplates in the decomposition range, indicating that the core of as-prepared nanowires was more stable than as-prepared nanoplates. This behavior might benefit from the hydrothermal process similar to the geological mineralization of bismutite. Given that C–BCO was prepared through the metathetical reaction between (BiO)NO_3_ and Na_2_CO_3_ solution at 55 °C, the total reaction time was relatively short. The rate of nucleation was so fast that intrinsic defects existed in (BiO)_2_CO_3_. For the nanowires, the hydrothermal process provided a homogeneous reaction environment for nucleation and growth of (BiO)_2_CO_3_ and guaranteed a high crystallinity similar to geological mineralization of bismutite. For Ca-BCO, doped Ca^2+^ ions distorted the lattice of (BiO)_2_CO_3_ and altered its lattice energy, resulting in a lower apparent activation energy compared to BCO [30]. These results were consistent with that of XRD. HRTEM images (Figure S4) clearly confirmed that the defects of BCO were located at the surface, whereas the stacking defects of Ca–BCO were located at the inner space due to the addition of Ca^2+^. Introducing Ca^2+^ ions into (BiO)_2_CO_3_ nanowires could improve the thermal stability of nanowires in terms of decomposition temperature. However, the decomposition activation energy of Ca–BCO was smaller than that of BCO. Distortion from doped Ca^2+^ ions in nanowires should thus accelerate the decomposition of (BiO)_2_CO_3_.

## 4. Conclusions

The effects of morphology and doped ions on the thermal stability of nanostructured (BiO)_2_CO_3_ were studied. Two decomposition zones existed in the TG curves of (BiO)_2_CO_3_ and Ca-doped (BiO)_2_CO_3_ nanowires prepared by hydrothermal synthesis, whereas only one decomposition zone was detected for the (BiO)_2_CO_3_ nanoplates from the metathetical reaction. Results show that structure, doped ions, and synthesis method had a significant effect on the thermal stability of nanostructured (BiO)_2_CO_3_. The decomposition temperature of nanostructured (BiO)_2_CO_3_ increased in the following order: Surface (BiO)_2_CO_3_ nanowires with defects < (BiO)_2_CO_3_ nanoplates < core of (BiO)_2_CO_3_ nanowires < core of Ca–(BiO)_2_CO_3_ nanowires. Kinetic analysis demonstrated that the apparent activation energies of the decomposition of surface (BiO)_2_CO_3_ nanowires with defects, (BiO)_2_CO_3_ nanoplates, core of (BiO)_2_CO_3_ nanowires, and core of Ca–(BiO)_2_CO_3_ nanowires were 118–123, 160–170, 230–270, and 210–223 kJ/mol, respectively. Doping of Ca^2+^ in (BiO)_2_CO_3_ nanowires improved the decomposition of (BiO)_2_CO_3_.

## Figures and Tables

**Table 1:** Thermogravimetric (TG) results of the samples obtained from different heating rates.

Sample	β/°C·min^−1^	1st Mass Loss Zone/°C	Peak Temperature/°C	Mass Loss/%	2nd Mass Loss Zone/°C	Peak Temperature/°C	Mass Loss/%
C-BCO	5				320.1–514.6	400.0	8.26
	10				324.9–543.9	417.9	8.33
	15				327.5–551.0	432.0	8.36
	20				328.0–563.5	436.0	8.40
BCO	5	193.3–367.8	276.3	1.47	367.8–624.8	434.8	4.23
	10	205.9–383.8	288.6	1.39	383.8–625.9	447.7	4.04
	15	211.2–387.2	296.2	1.41	387.2–635.2	453.7	4.10
	20	215.4–394.9	315.4	1.43	394.9–641.9	460.4	4.07
Ca-BCO	5	209.3–341.3	271.8	0.80	341.3–621.8	451.3	5.10
	10	211.5–355.5	285.6	0.81	355.5–637.0	464.6	5.13
	15	217.4–379.4	293.9	1.03	379.4–641.9	472.9	5.03
	20	213.6–368.6	297.6	0.69	368.6–650.6	479.1	5.07

**Table 2:** Apparent activation energies of nanostructured (BiO)_2_CO_3_ samples based on the Kissinger’s method.

Samples	1st Weight-Loss Zone		2nd Weight-Loss Zone	
	Ea/kJ/mol	R^2^	Ea/kJ/mol	R^2^
C-BCO			159.48	0.99123
BCO	118.69	0.9773	226.77	0.99363
Ca-BCO	122.84	0.9970	213.89	0.99928

**Table 3:** Apparent activation energy of nanostructured (BiO)_2_CO_3_ samples calculated by Kissinger–Akahira–Sunose’s methods in a range of α = 20–80%.

Conversion Rate/%	C-BCO		BCO		Ca-BCO	
	*E_a_*/kJ/mol	*R* ^2^	*E_a_*/kJ/mol	*R* ^2^	*E_a_*/kJ/mol	*R* ^2^
20	178.49	0.99794	246.71	0.99848	204.02	0.99699
30	169.32	0.9965	249.88	0.99914	207.59	0.99864
40	166.37	0.99656	253.81	0.99797	209.37	0.99905
50	163.72	0.99692	262.43	0.99618	213.22	0.9982
60	161.53	0.99729	276.72	0.99457	219.15	0.9977
70	159.92	0.99749	293.11	0.99522	226.79	0.99846
80	157.42	0.99784	334.49	0.99375	234.47	0.9975
Average	164.39 ± 4.94		257.91 ± 10.78 *		216.37 ± 6.84	

* The average apparent activity energy of as-prepared (BiO)_2_CO_3_ nanowires was calculated based on α = 20–60%.

## Supplementary Materials

The following are available online at https://www.mdpi.com/1996-1944/13/19/4287/s1, Figure S1: TG-DTG curves of (BiO)_2_CO_3_ nanoplates, Figure S2: TG-DTG curves of (BiO)_2_CO_3_ nanowires, Figure S3: TG-DTG curves of Ca-(BiO)2CO3 nanowires, Figure S4: TEM and HRTEM images of (BiO)_2_CO_3_ nanowires (a,b) and Ca-(BiO)_2_CO_3_ nanowires(c,d).
