# Supplementary material for: Thermal Decomposition of Nanostructured Bismuth Subcarbonate
Source: Materials (Basel). 2020 Sep 25;13(19):4287. doi: 10.3390/ma13194287 (PMC7579297; doi:10.3390/ma13194287)
Supplement: Supplementary file 1 [file materials-13-04287-s001.pdf]

Supplementary Information

Thermal Decomposition of Nanostructured Bismuth Subcarbonate

Su Sheng [1,2], Shengming Jin [1,2] and Kuixin Cui [1,2,*]

[1] School of Minerals Processing and Bioengineering, Central South University, Changsha 410083, China; shengsu0101@163.com (S.S.); shmjin@csu.edu.cn (S.J.)

[2] Key Laboratory for Mineral Materials and Application of Hunan Province, Central South University, Changsha 410083, China

* Correspondence:kuixin.cui@csu.edu.cn

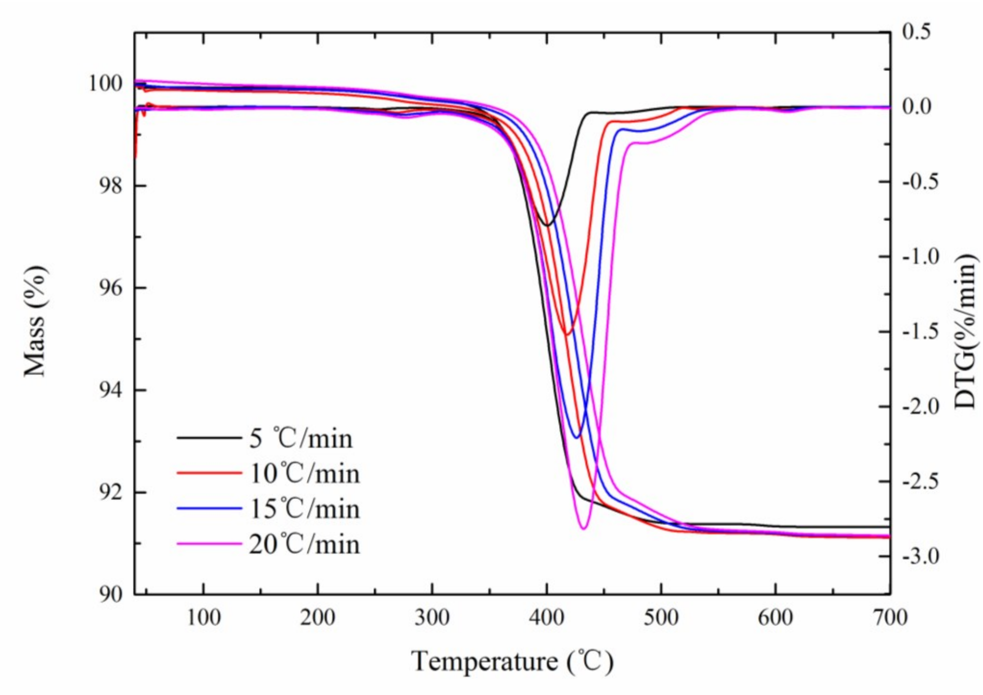

Figure S1. TG-DTG curves of $(BiO)_2CO_3$ nanoplates.

Figure S1 shows the TG-DTG curves of $(BiO)_2CO_3$ nanoplates at different heating rates. As shown in Figure S1, a distinct DTG peak, resulted from the thermal decomposition of $(BiO)_2CO_3$, was observed at about 400.0, 417.0, 432.0 and 426.0 °C for heat rate of 5 °C/min, 10 °C/min, 15 °C/min and 20 °C/min, respectively.

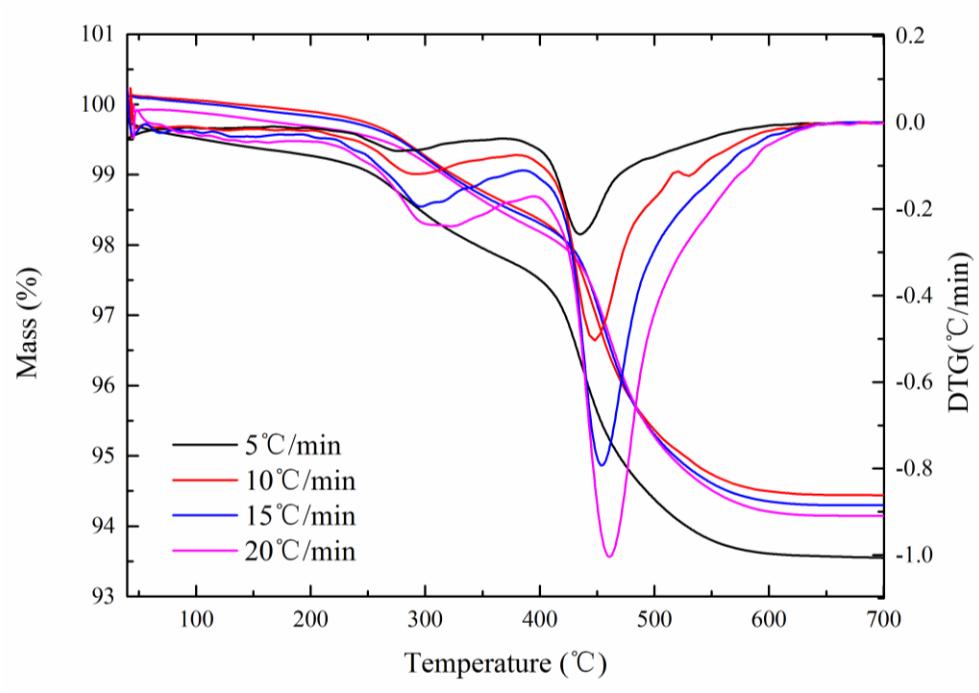

Figure S2. TG-DTG curves of $(BiO)_2CO_3$ nanowires.

Figure S2 shows the TG-DTG curves of $(BiO)_2CO_3$ nanowires at different heating rates. As shown in Figure S2, two distinct DTG peaks were observed at the temperature range of 200–380 °C and 380–600 °C, respectively.

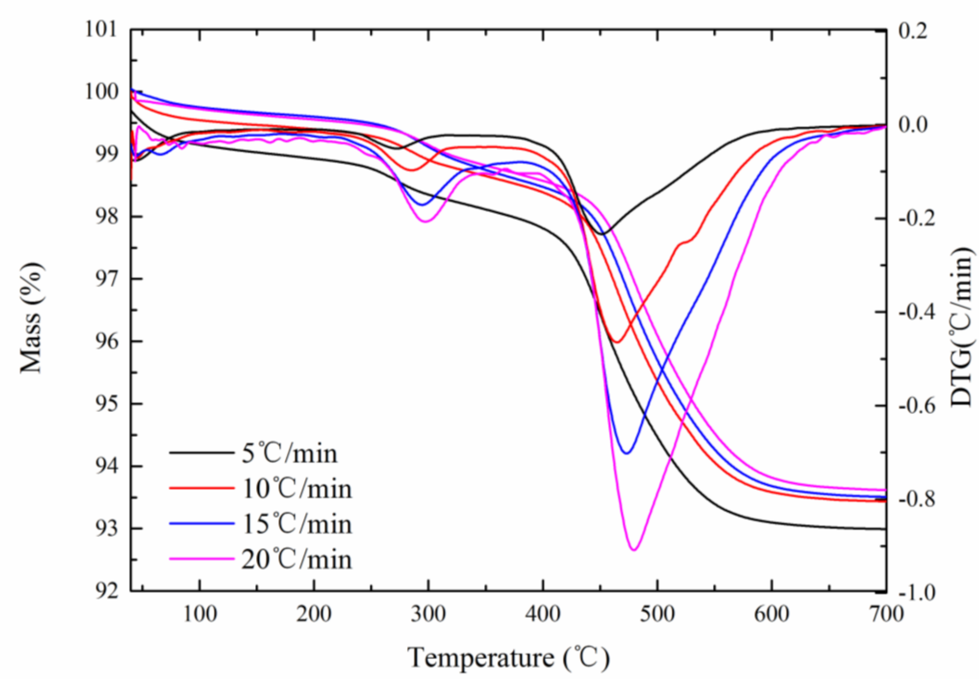

Figure S3. TG-DTG curves of Ca-$(BiO)_2CO_3$ nanowires.

Figure S3 shows the TG-DTG curves of Ca-doped $(BiO)_2CO_3$ nanowires at different heating rates. As shown in Figure S3, two distinct DTG peaks were observed at the temperature range of 200–380 °C and 380–600 °C, respectively.

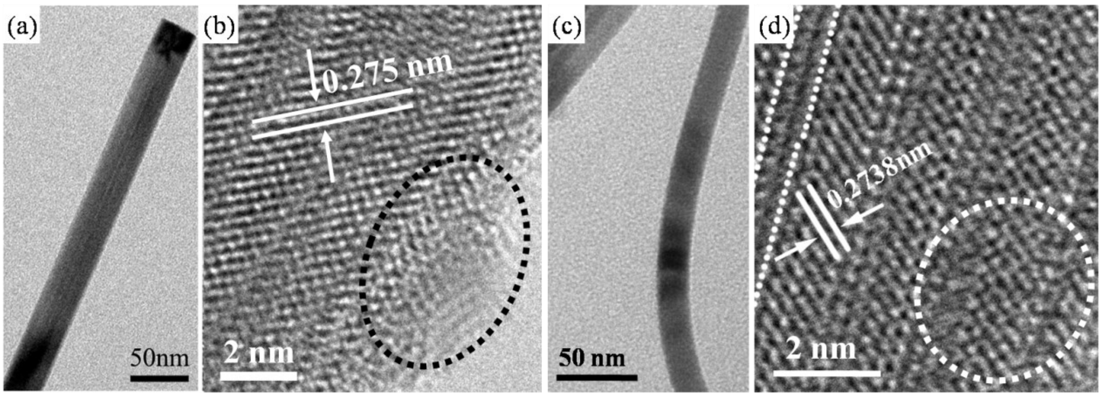

Figure S4. TEM and HRTEM images of $(BiO)_2CO_3$ nanowires (**a**,**b**) and Ca-$(BiO)_2CO_3$ nanowires(**c**,**d**).

Figure S4a and FigureS4b show the TEM and HRTEM image of as-prepared $(BiO)_2CO_3$ nanowire, respectively. It can be seen that the nanowires was about 30 nm in diameter. Figure S4b shows noticeable crystal lattice fringes and surface defects (showed in dash oval), indicating that the core of nanowires with good crystallinity were induced. The average distance between adjacent fringes was about 0.275 nm, consistent with (110) crystallographic planes of $(BiO)_2CO_3$. Similarly, the diameter of Ca-doped $(BiO)_2CO_3$ nanowires was 25 nm or so in Figure S4c and the crystal lattice fringes distance was 0.2738 nm in Figure S4d, corresponding to the (110) crystallographic planes of $(BiO)_2CO_3$. Moreover, a lot of defects (showed in dash area) could be observed in Figure S4d.
